# Rational Confinement of NiMo_6_ Polyoxometalates in a Single-Walled Carbon Nanotube: A High-Filling-Ratio Strategy for Enhanced Electrochemical Activity

**DOI:** 10.3390/mi17050583

**Published:** 2026-05-07

**Authors:** Kai Zhang, Zeling Yang, Chengxu Zhou, Xinwang Cao, Xiyuan Feng

**Affiliations:** 1 School of Textile Science and Engineering, Wuhan Textile University, Yangguang Road 1, Wuhan 430200, China; 2 Institute of Materials Science and Devices, School of Materials Science and Engineering, Suzhou University of Science and Technology, Suzhou 215009, China; 3 Co-Innovation Center of Efficient Processing and Utilization of Forest Resources, College of Materials Science and Engineering, Nanjing Forestry University, No. 159 Longpan Road, Nanjing 210037, China; 4 School of Microelectronics, Northwestern Polytechnical University, No. 1 Dongxiang Road, Chang’an District, Xi’an 710129, China

**Keywords:** carbon nanotube, confined encapsulation, NiMo_6_@SWCNT nanocomposite and enhanced OER performance

## Abstract

This study successfully developed an efficient one-dimensional confinement strategy to encapsulate polyoxometalate NiMo_6_ clusters densely and uniformly within the cavities of a single-walled carbon nanotube (SWCNT), constructing a unique core–shell NiMo_6_@SWCNT composite electrocatalyst. Comprehensive characterization including high-resolution transmission electron microscopy (HRTEM), energy-dispersive X-ray spectroscopy (EDS), X-ray diffraction (XRD), X-ray photoelectron spectroscopy (XPS), Raman spectroscopy, Fourier transform infrared spectroscopy (FTIR), and ultraviolet-visible absorption spectroscopy (UV-Vis) systematically confirmed the uniform dispersion and structural integrity of NiMo_6_ within the SWCNT channels. Key evidence encompasses: (1) EDS elemental mapping revealing high co-localization of Ni/Mo signals inside the lumens; (2) transmission electron microscopy (TEM) images confirming the effectiveness of the filling process. The composite achieved an exceptionally low overpotential of 308 mV to drive a current density of 10 mA cm^−2^ (significantly outperforming pure NiMo_6_ at 365 mV and pristine SWCNT at 519 mV), exhibited a remarkably low Tafel slope of 96.64 mV dec^−1^, possessed a high electrochemical active surface area (10.75 mF cm^−2^), and very low charge transfer resistance. Critically, it showed negligible current density decay during prolonged chronoamperometric operation over 35,000 s (>9.7 h). This work not only validates the confined encapsulation as a viable strategy for fabricating highly active polyoxometalate/carbon composites, but also elucidates that the performance enhancement stems from a “triple synergy”: the intrinsic catalytic activity of NiMo_6_, the highly conductive/mass-transport network provided by SWCNT, and the synergistic effects arising from the confined interface—namely stress regulation and electronic coupling. This insight provides a novel perspective for designing high-performance non-precious metal electrocatalysts.

## 1. Introduction

Polyoxometalates (POMs) are a class of metal–oxygen clusters with well-defined structures. Due to their rich redox activity, tunable composition, and high electron storage capacity, they exhibit great potential in the field of catalytic materials, particularly in the oxygen evolution reaction (OER) [1,2,3,4]. OER is a critical half-reaction in energy conversion and storage processes such as water electrolysis for hydrogen production and metal-air batteries. However, the design and development of highly efficient and stable OER catalysts remain a significant challenge [5,6]. Currently, OER electrocatalysts based on transition metal oxides and composite materials show good performance in terms of activity and stability but are limited in practical applications due to their high cost and complex synthesis processes. POMs, especially those containing transition metals such as nickel and molybdenum, like the Anderson-type NiMo_6_ clusters, demonstrate great potential for OER applications due to their unique structure and excellent electrochemical activity. These materials provide multiple potential active sites for the OER process with their rich redox centers and tight poly-metallic structure. However, similar to most OER electrocatalysts, POMs face challenges of insufficient exposure of active sites and poor structural stability during electrochemical processes. In particular, they are prone to structural degradation and dissolution in high-temperature or highly alkaline environments during the OER process, leading to a decline in cycling stability [7,8,9,10].

To address these issues, compositing POMs with carbon nanotube (CNTs) can enhance their catalytic performance and stability in OER. CNTs possess excellent conductivity, high specific surface area, and mechanical stability, providing efficient electronic transmission pathways and enhanced double-layer capacitance effects [11]. Additionally, serving as a support for POMs, CNTs can stabilize the POM structure and prevent aggregation and dissolution during electrochemical processes, thereby maintaining the stability of OER activity. Currently, the primary methods for combining POMs with CNTs include surface adsorption, layer-by-layer self-assembly, or simple blending. While these approaches can improve OER catalytic performance, the fixation and stability of POMs during long-term electrochemical cycling remain a critical issue. A more forward-looking strategy is to confine the active materials within the inner cavity of CNTs to construct a one-dimensional core–shell structure. This unique “filling” architecture can contribute to enhancing both the stability and activity of OER catalysts. The walls of the CNTs serve as a physical barrier, preventing the dissolution and loss of POMs in the electrolyte and avoiding the loss of active sites. Furthermore, the confined space may modulate the electronic structure and electron density of the active centers of POMs, optimizing their OER catalytic activity and selectivity. The one-dimensional conductive channels of carbon nanotube also ensure efficient electronic transmission, improving the reaction kinetics of the OER process [12].

Based on the above background, this paper proposes and realizes an innovative method for constructing a stable one-dimensional core–shell composite material by confining NiMo_6_ polyoxometalate (POM) clusters inside single-walled carbon nanotube (SWCNT) using an internal cavity filling strategy, specifically for oxygen evolution reaction (OER) catalysis. This study systematically characterizes the microstructure, chemical composition, and electronic state of the composite and comprehensively evaluates its electrochemical performance as an OER electrocatalyst. The results demonstrate that this “filled” structure not only significantly enhances the material’s stability over long-term electrochemical cycling but also achieves high activity and superior OER electrocatalytic performance through the synergistic effect between the carbon tubes and the POMs. This work provides new insights and experimental evidence for designing next-generation high-performance, long-life OER electrocatalysts.

## 2. Results and Discussion

### 2.1. Synthesis and Filling of NiMo_6_@SWCNT Composite Materials

**Figure 1:**
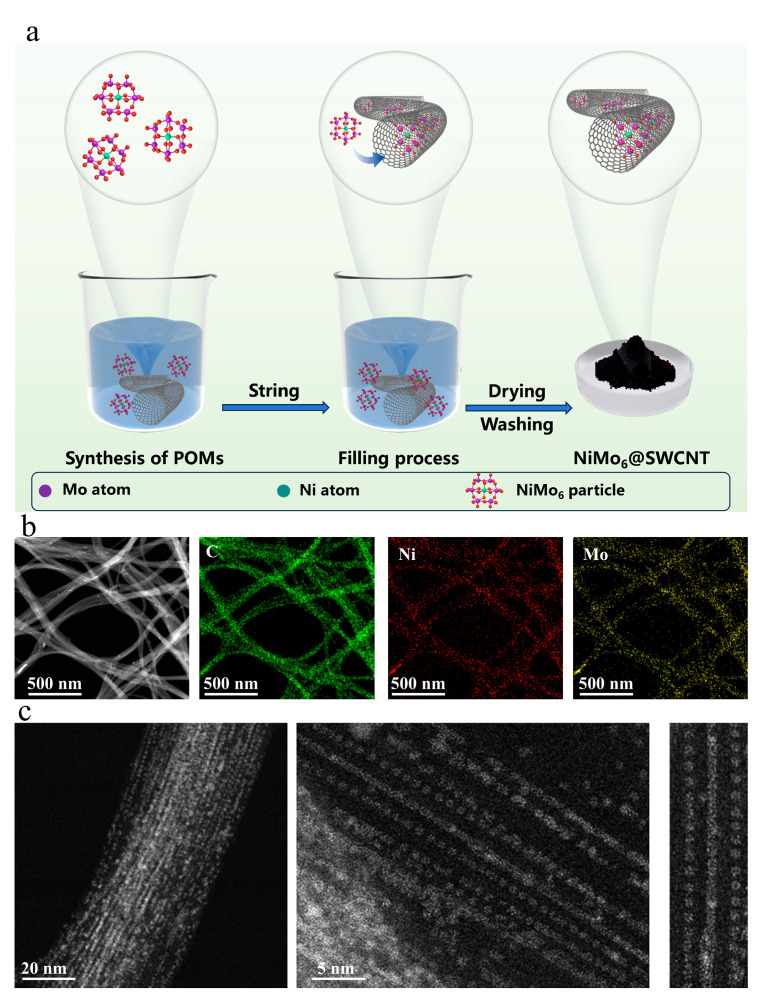
(**a**) Schematic illustration of the synthesis and filling process of NiMo_6_@SWCNT composite materials. (**b**) EDS elemental mapping of NiMo_6_@SWCNT composite materials, showing the distribution of C, Ni, and Mo elements. (**c**) HAADF-STEM images of NiMo_6_@SWCNT.

Figure 1a illustrates the synthesis of polyoxometalate NiMo_6_ clusters and their encapsulation into single-walled carbon nanotube cavities. The left section shows the formation of NiMo_6_ clusters where Ni, Mo and oxygen atoms assemble into polyoxometalate structures in a reaction vessel; the central part demonstrates the filling process with synthesized NiMo_6_ cluster solution and SWCNT dispersed in solvent where stirring facilitates gradual cluster migration into nanotube lumens; the right portion presents the dried and purified NiMo_6_@SWCNT composite [13]. Figure 1b presents high-angle annular darkfield scanning transmission electron microscopy (HAADF-STEM) images of the as-prepared NiMo6@SWCNT composite. At low magnification, NiMo6 is observed to be well-filled inside the carbon nanotube with high filling efficiency. The high-magnification images clearly reveal continuous and uniformly distributed NiMo6 clusters successfully encapsulated within the single-walled carbon nanotube. Meanwhile, the outer walls of the carbon nanotube remain clean, indicating the absence of residual coatings on the external surfaces. The EDS mapping results further corroborate the above conclusions (Figure 1c). The carbon signal (green) uniformly covers the entire nanotube region, indicating that the SWCNT structure remains intact. An obvious enrichment region of the nickel signal (red) is observed inside the nanotubes, confirming that the NiMo6 component has entered the nanotube cavities. The distribution pattern of the molybdenum signal (yellow) is highly consistent with that of nickel, also concentrating within the carbon nanotube, suggesting the coexistence of Mo and Ni and supporting the conclusion that the NiMo6 clusters are encapsulated as intact entities within the nanotube cavities [14].

### 2.2. Characterization and Analysis of Crystal Structure and Electronic States in the NiMo_6_@SWCNT Composite

**Figure 2:**
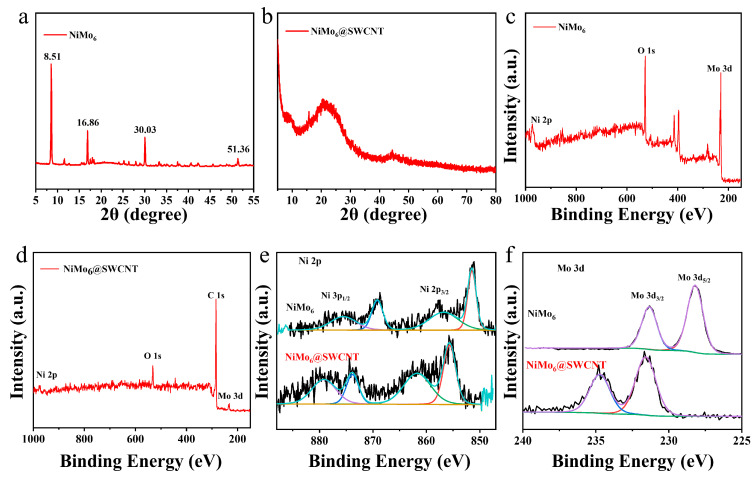
(**a**) XRD pattern of pristine NiMo_6_. (**b**) XRD patterns of NiMo_6_@SWCNT composite. (**c**) XPS survey spectrum of pristine NiMo_6_. (**d**) XPS survey spectrum of NiMo_6_@SWCNT composite. (**e**) High-resolution XPS spectrum of Ni 2p. (**f**) High-resolution XPS spectrum of Mo 3d.

XRD analysis was performed to confirm the crystal structure and phase composition of NiMo_6_@SWCNT composite. As shown in Figure 2a, the characteristic diffraction peaks of NiMo_6_ at 8.51°, 16.86°, 30.03°, and 51.36° are clearly detected in the composite, which are the typical peaks of Anderson-type polyoxometalates, demonstrating that NiMo_6_ clusters maintain their intact crystal structure after encapsulation [15]. The presence of the (002) peak of SWCNT at ~26.0° indicates that the structural integrity of SWCNT is well preserved. The weak intensity of NiMo_6_ peaks is attributed to the uniform dispersion of NiMo_6_ clusters inside the SWCNT lumens (Figure 1b), which is consistent with the TEM and EDS results.

High-resolution XPS spectra of pure NiMo_6_ showed distinct peaks of Mo 3d, Ni 2p, and O 1s. The Mo 3d spectrum exhibited two characteristic peaks at 229.8 eV (Mo 3d_5/2_) and 232.9 eV (Mo 3d_3/2_), consistent with the standard binding energy of Mo in Anderson-type POMs. The Ni 2p spectrum displayed two main peaks at 855.4 eV (Ni 2p_3/2_) and 873.9 eV (Ni 2p_1/2_), indicating the stable chemical state of Ni. The O 1s peak at 532.1 eV corresponded to the metal–oxygen bond (M–O) in NiMo_6_. The composite exhibited clear Mo 3d, Ni 2p, O 1s, and C 1s signals. The Mo 3d and Ni 2p peaks were consistent with those of pure NiMo_6_, confirming that NiMo_6_ was successfully incorporated into the composite without structural damage. The C 1s peak originated from SWCNT, further verifying the successful combination of NiMo_6_ and SWCNT. Deconvolution analysis of Ni 2p spectra showed that the Ni 2p_3/2_ and Ni 2p_1/2_ peaks of the composite were almost overlapping with those of pure NiMo_6_, indicating that the core electronic structure of Ni remained unchanged. Notably, the Ni 2p peaks of the composite slightly shifted to lower binding energy compared with pure NiMo_6_, which was attributed to the electronic interaction between NiMo_6_ and SWCNT. Similarly, the Mo 3d peaks of the composite also shifted to lower binding energy, which was associated with the interfacial electron transfer between SWCNT and NiMo_6_. These shifts confirmed the interfacial interaction between NiMo_6_ and SWCNT, providing evidence for the synergistic effect of the composite. XPS results demonstrated that the composite was composed of Mo, Ni, O, and C elements, without any impurity elements. The slight shift in Ni 2p and Mo 3d binding energy indicated the electronic interaction between NiMo_6_ and SWCNT, which laid a foundation for the enhanced electrocatalytic performance of the composite.

### 2.3. Spectral Characterization and Structural Analysis of NiMo_6_@SWCNT Composite Material

**Figure 3:**
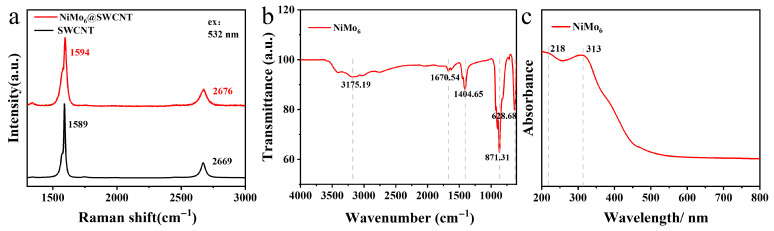
(**a**) Raman spectra of NiMo_6_@SWCNT and SWCNT (**b**) FT-IR Spectra and (**c**) UV-Vis Absorption Spectra of NiMo_6_.

Raman spectrum (Figure 3a) indicates that, compared to the typical G-band position of single-walled carbon nanotube (SWCNT) at 1589 cm^−1^, the G-band position for the filled sample (NiMo6@SWCNT) underwent a blue shift of approximately 5 cm^−1^ (moving to 1594 cm^−1^), along with a significant increase in peak intensity [16]. Additionally, the 2D-band position shifted from 2669 cm^−1^ to 2676 cm^−1^, also indicating a blue shift towards higher wavenumbers. These changes suggest some type of interaction between NiMo6 and SWCNT. Based on the high-angle annular dark field-scanning transmission electron microscopy (HAADF-STEM) images, since the NiMo6 clusters occupy the inner cavity of the carbon nanotube, they exert radial compressive stress on the tube walls, which could lead to the G-band blue shift from 1589 cm^−1^ to 1594 cm^−1^. Although a charge transfer effect may also contribute to this blue shift, further verification is still required [17].

Infrared spectroscopy (Figure 3b) provides direct evidence for the internal chemical bond vibrations of the NiMo_6_ molecular clusters. The broad absorption trough at 3175.19 cm^−1^ strongly points towards O–H stretching vibrations, very likely originating from water molecules adsorbed on the surface of the NiMo_6_ clusters or from hydroxyl (–OH) groups present within the structure, indicating a degree of hygroscopicity in ambient air [17]. The absorption trough at 1670.54 cm^−1^ may correspond to the H–O–H bending vibration of water molecules or C=O stretching vibrations; the presence of the latter would suggest partial oxidation or the adsorption of organic species on the surface. The absorption band near 1404.65 cm^−1^ is commonly assigned to asymmetric metal–oxygen (M–O) stretching vibrations or O–H bending vibrations [18]. Absorption troughs at lower wavenumbers, such as 871.31 cm^−1^ and 628.68 cm^−1^, fall within the characteristic region for polyoxometalate (POM) compounds. They likely correspond to stretching vibrations of Mo–O–Mo bridge bonds or bending modes of Mo=O terminal groups [19,20]. Collectively, these characteristic features confirm that the encapsulated material possesses a structure typical of NiMo_6_ polyoxometalate clusters, with IR fingerprints consistent with those reported in the literature for analogous POM compounds.

Ultraviolet-visible (UV-Vis) absorption spectroscopy (Figure 3c) elucidates the electronic transition properties of the NiMo_6_ clusters. The strong primary absorption peak appearing at 313 nm is typically identified as characteristic of ligand-to-metal charge transfer (LMCT) transitions in polyoxometalates (O → M). This involves a transition of lone-pair electrons from the oxygen ligands to unoccupied d-orbitals of the transition metal atoms (Mo or Ni). The position and intensity of this peak directly reflect the electronic energy level structure and the optical band gap of the NiMo_6_ clusters. The shoulder peak at 218 nm could correspond to higher-energy charge transfer transitions or other allowed electronic transitions within the cluster. These distinct UV absorption signatures indicate that the NiMo_6_ clusters maintain their molecular structural integrity in solution or dispersed state and possess a well-defined, readily detectable electronic structure.

The observed changes in the Raman spectra (blue shift in the G-band and peak intensity enhancement) provide direct evidence for the interaction between NiMo_6_ and SWCNT. This interaction is likely due to the unique interfacial effects arising from the confinement of the filler within a one-dimensional nanoscale cavity. FT-IR unequivocally confirms the chemical identity of the encapsulated species as the NiMo_6_ polyoxometalate cluster and suggests that its surface chemistry (e.g., adsorbed water) could influence the interfacial properties of the composite material. UV-Vis spectroscopy definitively establishes the inherent optical activity of NiMo_6_, with its absorption edge situated in the UV region. This implies that the composite material may exhibit optoelectronic properties governed by both the carbon nanotube and the encapsulated NiMo_6_ cluster. The data from these complementary spectroscopic techniques mutually corroborate the conclusion that NiMo_6_ was successfully encapsulated within the cavities of the SWCNT [21].

### 2.4. Electrochemical Performance and Stability Analysis of NiMo_6_@SWCNT Composite Material

To systematically evaluate the electrocatalytic oxygen evolution reaction (OER) performance of single-walled carbon nanotube (SWCNT), NiMo_6_ clusters, and the NiMo_6_@SWCNT composite, a series of electrochemical measurements were conducted in 1.0 M KOH electrolyte using a standard three-electrode system.

**Figure 4:**
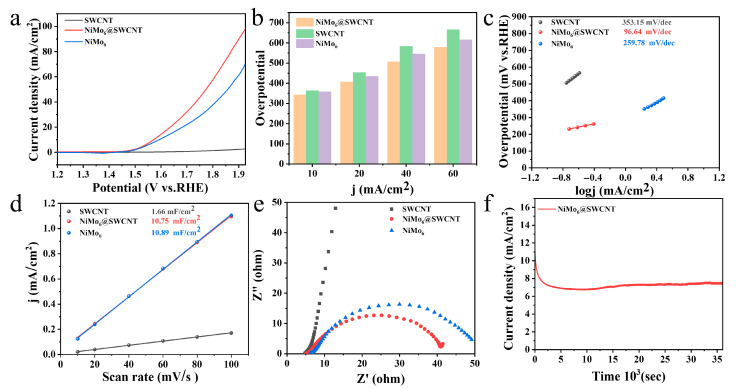
Electrochemical oxygen evolution performance of the catalysts. (**a**) Linear sweep voltammetry curves. (**b**) Overpotential values at different current densities. (**c**) Tafel slope plots. (**d**) Double-layer capacitance results. (**e**) Nyquist plots from electrochemical impedance spectroscopy. (**f**) Long-term stability test of NiMo_6_@SWCNT.

As shown in Figure 4a, the linear sweep voltammetry (LSV) curves clearly demonstrate that the NiMo_6_@SWCNT composite achieves the highest current density across the entire potential range, outperforming both pristine SWCNT and bare NiMo_6_ clusters. Figure 4b reveals that the NiMo_6_@SWCNT composite exhibits substantially lower overpotentials at all tested current densities compared to pure SWCNT, with performance comparable to or even exceeding that of NiMo_6_ alone. Figure 4c presents the Tafel slope analysis, which further confirms that the NiMo_6_@SWCNT composite possesses the smallest Tafel slope of 96.64 mV/dec, markedly lower than those of pure SWCNT (353.15 mV/dec) and bare NiMo_6_ (259.78 mV/dec), indicating significantly accelerated OER kinetics.

As shown in Figure 4d, electrochemical double-layer capacitance measurements verify that NiMo_6_ and NiMo_6_@SWCNT exhibit much larger electrochemical active surface areas than SWCNT. The Cdl value of NiMo_6_ is 10.89 mF/cm^2^ and that of NiMo_6_@SWCNT is 10.75 mF/cm^2^, in contrast to only 1.66 mF/cm^2^ for SWCNT. The similar Cdl values between NiMo_6_ and NiMo_6_@SWCNT indicate that the composite maintains abundant and highly accessible active sites for the oxygen evolution reaction. The enhanced OER activity of NiMo_6_@SWCNT does not solely rely on the improvement of electrochemically active surface area. It arises from the synergistic effect between sufficient active sites provided by NiMo_6_ and the highly conductive SWCNT network that facilitates rapid electron transfer and mass transport. Such a synergistic effect significantly accelerates the OER kinetics and contributes to the superior electrocatalytic performance even with similar electrochemically active surface area.

Electrochemical impedance spectroscopy (EIS) was performed at a DC bias potential of 1.5 V vs. RHE over a frequency range from 10^6^ Hz to 0.01 Hz with an AC amplitude of 5 mV, as displayed in Figure 4e. Among the three samples, the NiMo_6_@SWCNT composite exhibits the smallest charge-transfer resistance, confirming that the conductive SWCNT network effectively enhances electron transfer efficiency.

Finally, chronoamperometric testing, shown in Figure 4f, verifies the excellent long-term stability of the NiMo_6_@SWCNT composite, with negligible current decay observed over 35,000 s of continuous operation. Collectively, these electrochemical results demonstrate that the NiMo_6_@SWCNT heterostructure achieves superior OER activity and stability, attributable to the synergistic effect between the highly active NiMo_6_ sites and the high-conductivity SWCNT support, which facilitates accelerated charge transfer.

## 3. Conclusions

This study successfully developed an efficient encapsulation strategy, enabling the dense and uniform filling of polyoxometalate NiMo_6_ clusters into the cavities of single-walled carbon nanotube (SWCNT), precisely constructing a core–shell NiMo_6_@SWCNT nanocomposite. High-resolution transmission electron microscopy (TEM) and energy-dispersive X-ray spectroscopy (EDS) mapping clearly demonstrated smooth and intact nanotube walls with uniformly dispersed nanoparticles, where Ni and Mo elements showed precise co-localization. HAADF-STEM confirmed the effectiveness of the encapsulation, with NiMo_6_ clusters accurately filling the inner cavities of the carbon nanotube. High-resolution X-ray photoelectron spectroscopy (XPS) analysis consistently detected a negative shift of approximately 1–2 eV in the binding energies of the Ni 2p and Mo 3d orbitals. This finding complements the observed ~5 cm^−1^ blue shift and enhanced peak intensity of the SWCNT G-band in Raman spectra; these features collectively reveal the interactions between the carbon nanotube and NiMo_6_ clusters.

This composite material exhibits outstanding electrocatalytic performance for the oxygen evolution reaction (OER), with a significantly reduced overpotential of 308 mV at 10 mA/cm^2^ (far superior to pristine SWCNT at 519 mV and pure NiMo6 at 365 mV). It also demonstrates an exceptionally low Tafel slope of 96.64 mV/dec (substantially better than pure NiMo6 at 259.78 mV/dec and pristine SWCNT at 353.15 mV/dec), along with a large electrochemical active surface area (10.75 mF/cm^2^) and ultra-low charge transfer resistance. Notably, during long-term chronoamperometric stability testing that exceeded 35,000 s (>9.7 h), its performance showed nearly negligible degradation, indicating excellent durability. The source of this superior integrated performance lies in the synergistic triple advantages: the high intrinsic activity of NiMo_6_ clusters, the efficient conductive/mass transport network provided by SWCNT, and the unique interfacial cooperation (stress effects and electronic coupling) fostered within the nanoconfined environment.

## 4. Methods

### 4.1. Synthesis of NiMo_6_

Dissolve (NH_4_)_6_Mo_7_O_24_·4H_2_O (5 g, 4.2 mmol) in 80 mL of boiling deionized water with vigorous stirring (1000 rpm). Separately, dissolve NiSO_4_·5H_2_O (0.79 g, 3.0 mmol) in 20 mL deionized water. Add the metal salt solution dropwise (3 mL/min) into the boiling molybdate solution. After the formation of a turbid mixture, maintain boiling under stirring for 30 min. Cool naturally to 40 °C and collect precipitated solid by hot filtration through a #4 glass frit (Shanghai Aladdin Biochemical Technology Co., Ltd., Shanghai, China). Purify by twice recrystallization: Redissolve crystals in deionized water (20 mL at 90 °C) followed by slow cooling to room temperature. Vacuum-dry the final colorless crystals at 40 °C for 12 h.

### 4.2. Synthesis of NiMo_6_@SWCNT Composites

The NiMo_6_@SWCNT composite was prepared as follows: H_4_[NiMo_6_O_24_]·6H_2_O crystals were accurately weighed to prepare an aqueous solution at 5 mg/mL concentration, filtered through a 0.22 μm microporous membrane for later use; an appropriate amount of single-walled carbon nanotube (diameter 1.2 ± 0.3 nm, length 5–30 μm) was pre-dispersed by probe sonication (750 W, 10 s on/5 s off duty cycle) in deionized water (resistivity ≥ 18.2 MΩ·cm) to form a stable 0.5 mg/mL colloidal dispersion; both precursors were mixed at a NiMo_6_: SWCNT mass ratio of 10:1 in a glass reactor and continuously stirred at 600 rpm on a thermostatic magnetic stirrer for 12 h, maintaining temperature at 25 ± 1 °C with complete light exclusion (using dark amber reactors wrapped in aluminum foil); the resulting mixture was vacuum-filtered through a polytetrafluoroethylene (PTFE) membrane (0.2 μm pore size, φ = 50 mm) with three cycles of rinsing using 100 mL deionized water followed by 20 mL anhydrous ethanol, and finally vacuum-dried at 50 °C (−0.1 MPa) for 4 h to obtain a dark green-black solid composite.

### 4.3. Electrochemical Measurements

All electrochemical measurements were performed on a CHI 760E electrochemical workstation (Shanghai Chenhua Instrument Co., Ltd., Shanghai, China) at room temperature using a standard three-electrode system. The electrolyte employed in all tests was 1.0 M KOH aqueous solution. The working electrode was prepared by dispersing 5 mg of catalyst powder in a mixed solution containing 950 μL of ethanol, and 50 μL of 5 wt% Nafion binder. The mixture was sonicated for 30 min to obtain a homogeneous catalyst ink, after which 20 μL of the ink was loaded onto a polished glassy carbon electrode with a diameter of 5 mm and dried naturally at room temperature. A platinum sheet was used as the counter electrode, and a Hg/HgO electrode was employed as the reference electrode. All measured potentials were calibrated to the reversible hydrogen electrode (RHE) for subsequent data analysis.

Linear sweep voltammetry (LSV) curves were recorded in the potential range of 1.0–1.8 V vs. RHE at a scan rate of 5 mV/s. All LSV data were subjected to 95% iR compensation to eliminate the interference of solution resistance. Tafel slopes were derived from the corresponding LSV curves based on the classic Tafel equation, so as to quantitatively analyze the oxygen evolution reaction kinetics.

The electrochemical double-layer capacitance (Cdl) was determined using cyclic voltammetry (CV) in a non-Faradaic potential window of 0.31–0.41 V vs. RHE. CV curves were collected at multiple scan rates ranging from 10 to 100 mV/s. The current density at a fixed intermediate potential was linearly fitted against the scan rate, and the slope of the fitted straight line was defined as the Cdl value, which was used to evaluate the electrochemically active surface area of each sample.

Electrochemical impedance spectroscopy (EIS) was carried out to investigate the charge transfer behavior of the catalysts. The tests were performed at a direct current (DC) bias potential of 1.5 V vs. RHE, with an alternating current (AC) amplitude of 5 mV and a frequency range from 10^6^ Hz to 0.01 Hz. The charge transfer resistance was obtained by fitting the corresponding Nyquist plots.

Long-term stability was evaluated using the chronoamperometry method. The test was conducted at a constant applied potential for a continuous duration of 35,000 s, and the current retention was recorded in real time to assess the operational stability of the catalyst.

### 4.4. Characterizations

In this work, to evaluate thermal stability and composition changes, we first performed thermogravimetric analysis in air using a NETZSCH TG 209 F1 instrument (NETZSCH Scientific Instruments Trading (Shanghai) Ltd., Shanghai, China). A heating rate of 15 °C min^−1^ was used, and the TG curve was obtained in the test range of 15~900 °C. The differential thermogravimetry (DTG) curve was calculated through the first derivative of the TG curve. For the observation of the sample’s microstructure and morphology as well as the analysis of elemental distribution, we utilized a spherical aberration corrected transmission electron microscope (AC-TEM, Tecnai G2F20) and combined it with X-ray energy dispersive spectroscopy (EDS) (Thermo Fisher Scientific Inc., Shanghai, China) for detailed analysis. A cold field emission scanning electron microscope (SU 8600) from HITACHI (HITACHI.Ltd., Ibarakiken, Japan) was employed, featuring an electron beam resolution of 0.6 nm at 15 kV and 0.7 nm in 1 kV deceleration mode. Before testing, the carbon nanotube fiber sample was stretched and attached to a conductive adhesive. The PHI 5000 Versaprobe II X-ray photoelectron spectroscopy (XPS) from ULVCA-PHI in Nanjing China was also used for XPS testing. The Al Kα ray source (hv = 1486.6 eV) was utilized with a working voltage of 25 V. For the characterization of the sample, an InVia Qontor confocal Raman microscope from Renishaw in the London UK was used. Under the excitation of a 532 nm He-Ne laser, the spot diameter for Raman scattering measurement was 4 μm, and the output power was 200 μW.

## Data Availability

The original contributions presented in this study are included in the article. Further inquiries can be directed to the corresponding authors.
